# Addition of Vegetable Oil to Improve Triterpenoids Production in Liquid Fermentation of Medicinal Fungus *Antrodia cinnamomea*

**DOI:** 10.3390/jof7110926

**Published:** 2021-10-31

**Authors:** Linghui Meng, Biaobiao Luo, Yang Yang, Mohammad Omar Faruque, Jiuliang Zhang, Xiaohua Li, Xuebo Hu

**Affiliations:** 1 Laboratory of Natural Medicine and Molecular Engineering, College of Plant Science and Technology, Huazhong Agricultural University, Wuhan 430070, China; menglinghui@webmail.hzau.edu.cn (L.M.); zoudian@webmail.hzau.edu.cn (B.L.); life333@webmail.hzau.edu.cn (Y.Y.); lixiaohua2007@hotmail.com (X.L.); 2 Ethnobotany and Pharmacognosy Laboratory, Department of Botany, University of Chittagong, Chittagong 4331, Bangladesh; omf@cu.ac.bd; 3 Department of Food and Nutrition, College of Food Science and Technology, Huazhong Agricultural University, Wuhan 430070, China; zjl_ljz@mail.hzau.edu.cn

**Keywords:** *Antrodia cinnamomea*, submerged mycelium, biomass, triterpenoids, liquid fermentation

## Abstract

The liquid fermentation of *Antrodia cinnamomea* is a promising alternative source for fungus production compared to the wildly grown fruiting body. Elicitation is a strong tool to enhance the productivity in microbial cells to obtain more compounds of interest. In this study, in order to improve the fungus growth and its terpenoids production, various vegetable oils were added in the fermentation broth of *A. cinnamomea*. It was found that corn oil from a group of vegetable oils exhibited the best effect on the biomass and triterpenoid content. After optimization, the initial addition of 1% (*v*/*v*) corn oil plus the inoculation of 10% (*v*/*v*) mycelia led to a maximum triterpenoid yield (532.3 mg L^−1^), which was increased as much as fourfold compared to the blank control. Differential transcriptome analysis demonstrated that corn oil significantly enriched several metabolic pathways including glycolysis/gluconeogenesis, propanoate metabolism and transmembrane hydrophobins. The enriched pathways interacted with deferentially expressed genes (DEGs) induced by corn oil treatment. Our research provides a potential strategy for the large production of triterpenoids by the improved fermentation of *A. cinnamomea*.

## 1. Introduction

*Antrodia cinnamomea* is a precious, host-specific brown-rot fungus which is well known to contain diverse bioactive compounds with potential pharmaceutical activity. In particular, *A. cinnamomea* is rich in polysaccharides and produces specific ergostane triterpenoids, including Antcin A, C and K, zhankuic acids A, B, and C, and antrocamphin [1,2,3,4,5]. *A. cinnamomea* has been used as a medicinal mushroom and has a long history as a remedy for various diseases, including hypertension, inflammation, hepatitis B, cancers and alcohol intoxication [1,6,7,8,9].

However, it is difficult to obtain the required amount of fruiting bodies of *A. cinnamomea* from their natural habitat due to the slow growth rate and the rare host tree *Cinnamomum kanehirai* Hay [10]. An alternative source could be the mycelia instead of dried fruiting bodies, mainly derived from two types of solid and liquid fermentation [11]. Solid type is a slow process that is difficult to monitor, control, and scale-up while liquid type is easier to monitor and to control key operational parameters [12]. Growing mycelia of this fungus in a defined nutrient medium by liquid fermentation is a simple and fast strategy to produce fungal biomass and valuable bioactive metabolites [13]. However, the rapid nutrient depletion causes decreasing the rate of biosynthesis of various secondary metabolites [14]. For example, previous research indicated that the triterpenoid yield is relatively 2.3-fold low in submerged *A. cinnamomea* mycelia than in its fruiting body [15].

Elicitation is a strong tool to enhance the productivity in plant cell [16]. The common elicitors for the abiotic types are metal ions and inorganic compounds, and for the biotic types are cell wall components of fungi or bacteria as well as released chemicals by organism [15,17]. Several studies indicated that elicitation is also able to improve the liquid fermentation of *A. cinnamomea*. A previous study reported to increase the amount of biomass and triterpenoids contents in submerged *A. cinnamomea* mycelia by adding chitosan and CaCl_2_ [15]. Moreover, the multiple additions of coenzyme Q and p-hydroxybenzoic acid enabled the submerged *A. cinnamomea* mycelia synthesize antroquinonol [18]. Triterpenoid production is enhanced tenfold after the addition of citrus peel extract, which contains rich essential oil [19]. In addition, the stimulatory effects of lipids on the exo-biopolymer production and mycelial growth have been confirmed in many other fungi [20,21]. Vegetable oil of 99% fatty acids has a wide resource. Commonly, various fatty acids in vegetable oil are used as the structural block to build in cellular membrane [22], and they can also work as a carbon resource to provide energy via β-oxidation for organism survival [23]. Besides, fatty acids in vegetable oil act as a regulator to affect cell growth and metabolism [24]. Currently, addition of vegetable oils containing rich fatty acids is used to improve productivity of liquid fermentation with various fungi, and for instance, cordycepin production was enhanced in liquid static cultivation of *Cordyceps militaris* by adding vegetable oils as the secondary carbon source [25]. The effect of vegetable oil on the production of mycelial biomass and polysaccharides was investigated in submerged culture of *Grifola frondosa* [26]. Cephamycin C production was also increased using soybean oil as the sole carbon source in *Streptomyces* mycelia [14]. Nonetheless, to the best of our knowledge, there is no report yet on improving the liquid fermentation of *A. cinnamomea* by the addition of vegetable oil.

Hence, six common vegetable oils with different composition were tested to improve the liquid fermentation of *A. cinnamomea* in this study. The effect of time and dosage of addition on mycelial biomass and triterpenoid content were determined. Moreover, to explore the molecular mechanism, differences of transcriptome expression pattern induced by addition of vegetable oil was also investigated. The outcome of this study would explore new insights for improving the production of bioactive components from this mushroom.

## 2. Materials and Methods

### 2.1. Chemicals

Corn oil and rape oil were purchased from Yihai Kerry Food Marketing Company (Beijing, China). Linseed oil was purchased from Greeno Co., Ltd. (Beijing, China). Palm oil was purchased from Angel Yeast Company (Yichang, China). Peanut oil was purchased from Luhua Group Co., Ltd. (Laiyang, China). Tea oil was purchased from Jinxin Agricultural Technology Development Co., Ltd. (Leiyang, China). Other reagents were bought from Sangon Biotech Co., Ltd. (Shanghai, China).

### 2.2. Microorganism and Media

The stock culture of *A. cinnamomea* strain AY378094 was cultured in potato-dextrose-agar (PDA) medium, containing extract of 200 g L^−1^ potato, 20 g L^−1^ glucose and 13 g L^−1^ agar. Inoculated plates were incubated at 28 °C for 30 days and then stored at 4 °C for pre-culture. Enlarge cultivation was performed with potato-dextrose (PD) medium. The fermentation medium was composed of 35 g L^−1^ glucose, 5 g L^−1^ tryptone, 5 g L^−1^ yeast extract, 0.5 g L^−1^ MgSO_4_·7H_2_O, and 1 g L^−1^ KH_2_PO_4_. The pH was adjusted to 6.0 by addition of 6 mol L^−1^ hydrochloric acid solution. 

### 2.3. Cultivation

40 agar discs (0.5 cm) covered by *A. cinnamomea* mycelium were obtained by 1 mL tip and then transferred into a 250 mL flask containing 100 mL PD medium. The mycelium was shaken at 28 °C with 150 rpm for 7 days. First, seed cultures were grown in 250 mL flasks containing 40 mL fermentation medium inoculated with 10 mL subcultures at 150 rpm and 28 °C for 5 days. Second, seed cultures were grown in 250 mL flasks containing 45 mL fermentation medium inoculated with 5 mL first seed cultures at 150 rpm and 28 °C for 2 days. Then, second seed were homogenized before inoculation. Fermentation was performed in the conditions as follows: 250 mL flasks containing 100 mL culture broth were put in 28 °C shaker at 150 rpm, and culture time was varied based on the different experimental plans.

For optimizing addition of corn oil, dosage of corn oil was varied from 1% to 4% (*v*/*v*), and addition time was set at 0 h, 24 h, 36 h, 48 h, 72 h, and 96 h during fermentation. For optimizing inoculation volume of seed cultures, inoculation volume was varied in the range of 5–15% (*v*/*v*).

### 2.4. Determination of Biomass and Triterpenoids

After fermentation, the fermentation broth was filtrated by filter paper in a Buchner funnel, followed by washing with double distilled water (ddH_2_O) to obtain mycelium. Obtained pellets of *A. cinnamomea* mycelia were then dried in oven at 50 °C. Biomass of submerged *A. cinnamomea* mycelia was determined using an electronic balance.

Extraction of triterpenoid was done followed by previously published protocol [27]. Briefly, 100 mg mycelium powder was sonicated in 5 mL 50% (*v*/*v*) ethanol for twice (each time for 2 h). The supernatants were dried at 50 °C using a rotary evaporation. The residues were then suspended with 3 mL ddH_2_O and then extracted with 5 mL chloroform for twice. The chloroform layer was dried at 45 °C using a rotary evaporation. Precipitations were dissolved in 3 mL methanol.

The measurement of The triterpenoid concentration in extraction was followed by a previous method [28] with a slight modification. Briefly, after a 100 μL sample solution was heated to evaporate at 70 °C in a water bath, 300 μL fresh 5% (*w*/*v*) vanillin-acetic solution and 1mL sulfuric acid were added and then incubated at 70 °C for 20 min. The cooled solution was diluted to 5 mL with acetic acid. Sample operation except sample solution was performed in the control. The absorbance of solution at 550 nm was measured against the control using a spectrophotometer. The triterpenoid content in sample was determined using the standard oleanolic acid calibration curve in a range of 0.02–0.12 mg (Y = 4.0905X − 0.0054, r^2^ = 0.9961; X, concentration; Y, absorbance value). Triterpenoid yield was calculated based on values of biomass.

### 2.5. Transcriptome Sequencing and Analyses

The submerged *A. cinnamomea* mycelia in the optimal fermentation and the control were sampled at day 12 for the extraction of total RNA. For the Illumina HiSeq2500 sequencing, complementary DNA was synthesized using SMART technology as previously described [29] and sequenced using SMRT sequencing technology on the PacBio RSII platform. Using Hisat2, Illumina reads from different samples were mapped to the genome (https://ftp.ncbi.nlm.nih.gov/genomes/all/GCA/000/766/995/GCA_000766995.1_ASM76699v1/GCA_000766995.1_ASM76699v1_genomic.fna.gz, accessed on 16 June 2016) [30]. The statistical models for maximum likelihood and maximum a posteriori implemented in Cufflinks (version 1.1.0) were used for expression quantification and differential analysis. The abundances are reported as normalized fragments per kb of transcript per million mapped reads. If expression of gene differs between the control and the treatment with a fold change > 2 and a *p* value < 0.05 as calculated by Cufflinks, it is considered significantly differentially expressed.

## 3. Results

### 3.1. Effects of Various Vegetable Oils 

**Figure 1:**
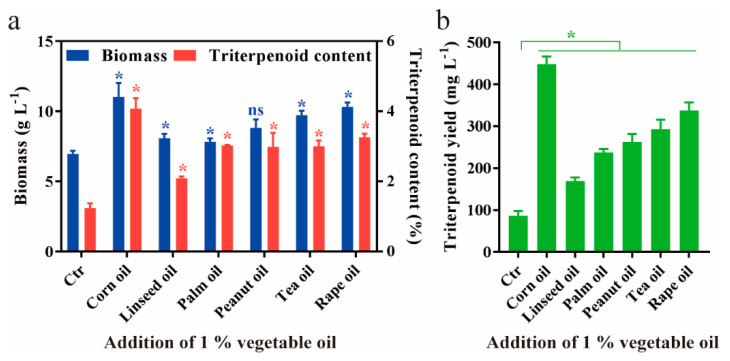
Effects of various vegetables oil on the fermentation of *A. cinnamomea*. Biomass and triterpenoid content (**a**), and triterpenoid yield (**b**), are quantitatively analyzed, respectively. The addition volume of various vegetable oils is 1% (*v*/*v*). The control has no vegetable oils. All fermentations are conducted at 28 °C for 12 days. All data are from triplicates (means ± SD). Asterisks denote (* *p* < 0.05) statistically significant differences between the treatment groups and the control; ns indicates no significant difference.

To improve the liquid fermentation *A. cinnamomea* mycelium, common vegetable oils, including corn oil, linseed oil, palm oil, peanut oil, tea oil and rape oil were added into fermentation medium while control was without any oil. Our study revealed that the biomass of submerged mycelia was significantly increased by adding vegetable oils (Figure 1a). For example, corn oil and rapeseed oil exhibited the highest biomass of mycelia (10~11 g L^−1^) followed by tea oil and olive oil whereas palm oil and linseed oil promoted lowest biomass, and peanut oil did not affect mycelium biomass. Likewise, triterpenoid content in submerged mycelia was also significantly increased (Figure 1a). Remarkably, corn oil exhibits the biggest boost to the triterpenoid content, reaching 4.1% (*v*/*v*), which exhibited an enhancement by 3.3-fold compared to the control. The other oils increased the triterpenoid content by only 1.7~2.6-fold, in the range of 2.1~3.3% (*v*/*v*). Consequently, of all oils tested, corn oil was demonstrated to be the most efficient on improving productivity, which promoted the highest triterpenoid yield of 448.4 mg L^−1^, which was 5.16-fold higher than the control (Figure 1b).

### 3.2. Optimization of Adding Corn Oil in Medium

**Figure 2:**
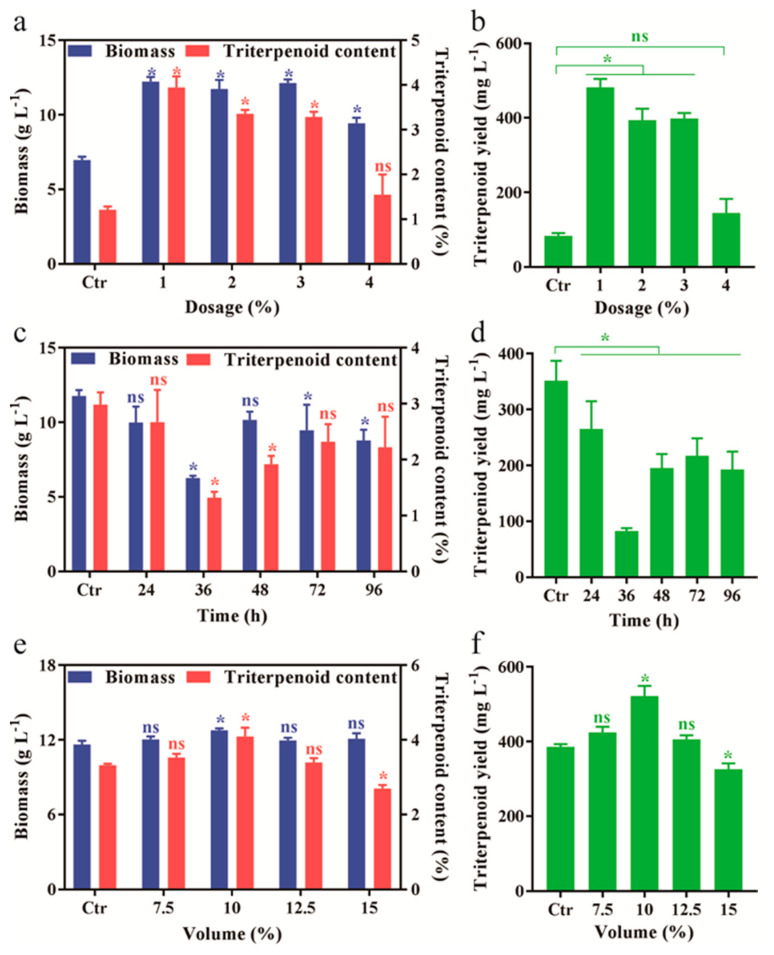
Optimization of the liquid fermentation of *A. cinnamomea* with corn oil. The effect of oil dosages (**a**,**b**), addition time (**c**,**d**), and inoculation volume of mycelia (**e**,**f**) on biomass and triterpenoid content, and triterpenoids yield, was quantitatively analyzed, respectively. The fermentation without oil, 0 h addition of 1% (*v*/*v*) corn oil and 5% (*v*/*v*) inoculation volume is considered as the control in a and b, c and d, and e and f, respectively. All fermentation was conducted at 28 °C for 12 days. All data were from triplicates (means ± SD). Asterisks denote (* *p* < 0.05) statistically significant differences between treatment groups and the control, ns indicates no significant difference.

For further improvement of fermentation with corn oil, effects of dosages and time for addition of corn oil on fermentation were investigated. As results showed in Figure 2a, the dosage of 1~3% (*v*/*v*) had significant positive impacts on mycelia biomass compared to the blank control of the fermentation without oil. However, the highest dosage of 4% (*v*/*v*) lead to a slight decrease on mycelia biomass. In addition, similar ranges of 1~3% (*v*/*v*) also produced significant effects on the triterpenoid content (Figure 2b). The maximum triterpenoid content was observed when *A. cinnamomea* mycelia were exposed to 1% corn oil. Also, 1% corn oil was proved to be the best addition dosage for triterpenoid yield while the high dosage of 4% had no effect (Figure 2b). Subsequently, the optimal time for adding corn oil was determined. The impacts of time variation on the biomass presented a gradually declining trend before 36 h, and then the mycelial biomass was increasing and exhibited relative constant in the range of 48~96 h. (Figure 2c). Interestingly, at the beginning time of the addition of corn oil (0 h), highest level of biomass was produced. In contrast, the varied times showed the similar effects to the triterpenoid content in submerged mycelia (Figure 2c) and the maximum triterpenoid yield was also obtained at the 0 h (Figure 2d) and hence, these two conditions were used for the optimal fermentation.

Before the optimal fermentation of *A. cinnamomea* mycelia, we also investigated the effect of the inoculation volume of seed cultures. The results showed that the inoculation of 10% (*v*/*v*) slightly increased mycelial biomass as well as significantly improved triterpenoid content (Figure 2e). Consequently, when 10% (*v*/*v*) second seed culture was inoculated in the medium, the triterpenoid yield (Figure 2f) was maximized. Then the inoculation of 10% (*v*/*v*) was used in the next optimal fermentation.

### 3.3. Optimal Fermentation with Corn Oil 

**Figure 3:**
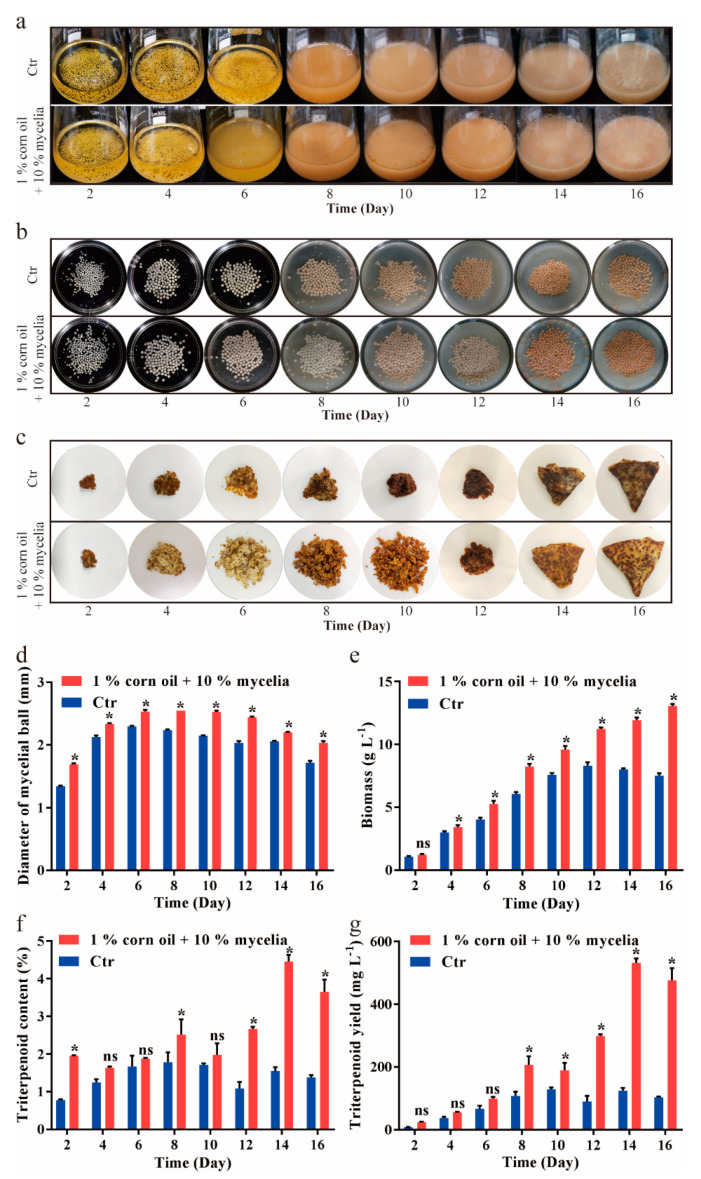
Change of fermentation broth in the optimal fermentations. Fermentation broth (**a**), mycelial ball (**b**) and dried mycelial pellet (**c**), was presented, respectively. During the optimal fermentations for 16 days, diameter of mycelial ball (**d**), biomass (**e**), triterpenoid content (**f**) and triterpenoids yield (**g**), was quantitatively determined, respectively. The control was the fermentation without oil. All data are from triplicates (means ± SD). Asterisks denote (* *p* < 0.05) statistically significant differences between the treatment groups and the control, respectively; ns indicates no significant difference.

Based on results above, the optimal liquid fermentation of *A. cinnamomea* (the initial addition of 1% (*v*/*v*) corn oil and inoculation of 10% (*v*/*v*) was performed for 16 days, and the liquid fermentation without oil addition was used as the control. Morphological changes of submerged *A. cinnamomea* mycelia were presented in Figure 3. The fermentation broth with oil started to become muddy at day six while the control kept clear before day 6 (Figure 3a). Moreover, corn oil seems to stop the earlier color change of aggregated mycelial balls (Figure 3b). In the fermentation with corn oil, mycelia balls began to turn red at day 10 while this happened at day eight in the control. The dried mycelia provided a visible sight to the changing biomass (Figure 3c). At day 16, the dried mycelia pellet in the fermentation with corn oil was obviously larger than the control.

In the fermentation processes, corn oil promoted the aggregation of *A. cinnamomea* mycelia, and as a result, the mycelial ball in the optimal fermentation was more than that of the control (Figure 3d). Moreover, the addition of 1% (*v*/*v*) corn oil resulted continuous increasing mycelia until the fermentation was completed. However, the mycelial biomass in the control began to decrease at day 12 (Figure 3e). The optimal fermentation resulted in the highest mycelial biomass of 13.00 g L^−1^, which was increased by 1.57-fold compared to the control. The changing of triterpenoid content exhibited the intermittent trends, which occurred to two peaks at day eight and day 14, respectively (Figure 3f). The highest level of triterpenoid content was reached at day 14 in the optimal fermentation while for the control, it was at day 8 (Figure 3f). Similarly, the optimal addition of corn oil promoted the triterpenoid content, increasing to a highest level of 4.46% (*w/w*), which was 2.5-fold more than the control. The changes of triterpenoid yield presented the similar trends like triterpenoid content (Figure 3g). Eventually, triterpenoid yield in optimal fermentation reached the maximum level of 532.3 mg L^−1^ at day 14, which was increased by 4.11-fold compared to the control.

### 3.4. Differential Transcriptome Analysis 

**Figure 4:**
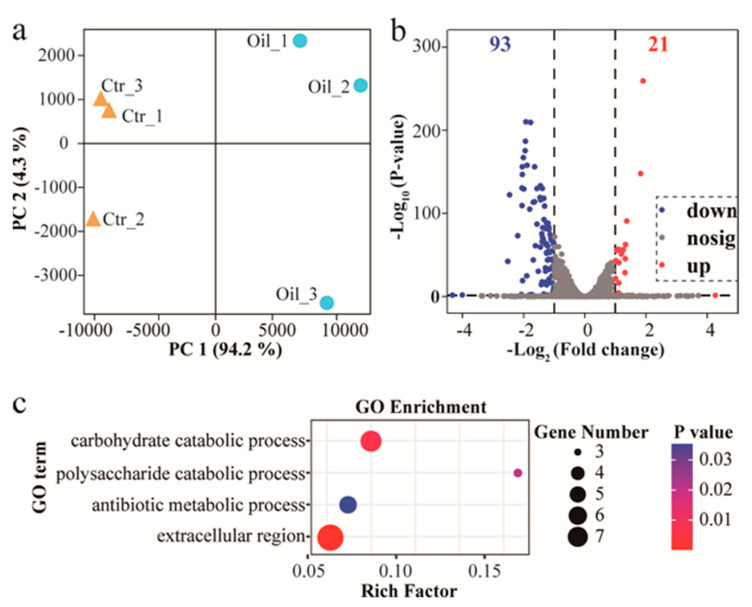
A plot of PCA analysis between samples (**a**), a volcano of DGEs (**b**), and a plot of enriched GO terms (**c**) was described, respectively.

To better understand the molecular mechanism of observed results, differential transcriptome analysis was performed on samples of submerged *A. cinnamomea* mycelia. Correlation between replicates was also indicated by the principal component analysis (PCA), which also showed the overall variation in gene expression for all analyzed samples (Figure 4a). PC1 and PC2 contribute 94.2% and 4.3% to the total variation, respectively. Differently expressed genes (DEGs) were identified from the RNA-Seq data (Table S1). A total of 114 DEGs (including 21 up- and 93 down-regulated DEGs) were identified (Figure 4b).

To explore the associated functions of DEGs in treated *A. cinnamomea* mycelia, gene ontology (GO) functional annotation was used to perform enrichment analysis and classifications. Enrichment results (Figure 4c) indicated that enriched 3 GO terms were significantly associated with the biological processes module, including “carbohydrate catabolic process”, “polysaccharide catabolic process” and “antibiotic metabolic process”. In contrast, enriched cellular component terms were significantly associated with only two GO terms, “extracellular region” and “hydrolase activity acting on glycosyl bonds”, were significantly enriched in cellular component module and molecular functions module, respectively.

**Figure 5:**
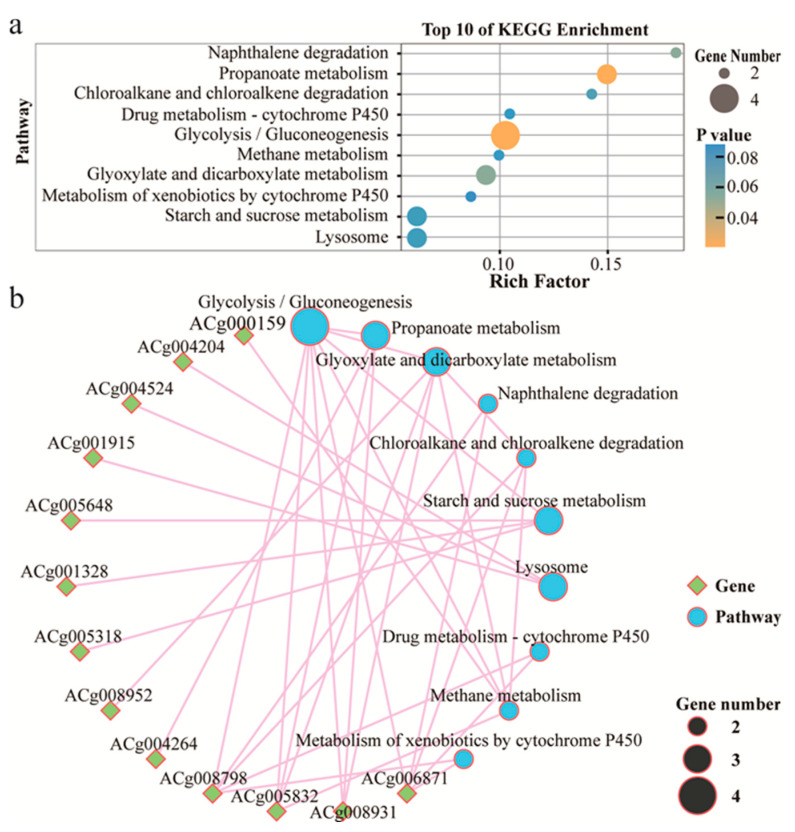
(**a**) Top 10 pathways of KEGG enrichment were detailed. (**b**) A network map of enriched pathways and their DGEs was descripted.

To identify the biological pathways that are active in submerged *A. cinnamomea* mycelia, we mapped DEGs to the reference of canonical pathways in the Kyoto Encyclopedia of Genes and Genomes (KEGG) database. In total, 21 DEGs were assigned to 36 KEGG pathways. Remarkably, 4 and 3 DEGs were significantly enriched in glycolysis/gluconeogenesis and propanoate metabolism, respectively (Figure 5a). The network of enriched pathways and their DEGs shown that glycolysis/gluconeogenesis and propanoate metabolism interacted with other pathways (Figure 5b).

## 4. Discussion

The main ingredients of vegetable oils are 95~96% (*w*/*w*) triglycerides, whose molecular structure, including the length of carbon chain and the ratio of unsaturated fatty acids, could significantly affect mycelial growth. Moreover, triglycerides are a type of lipid, which are the building blocks of a cell’s envelope and the cell membrane, and as signals, some of them also play a regulatory role and decisively influence cell growth [31]. The molecular structures of fatty acids in vegetable oils also determine the extent of stimulation or suppression in metabolite production [21,32].

The tested oils present a series of varied compositions that make an excuse for their distinct performances on the fermentation of *A. cinnamomea* mycelia. Among all oils tested, the addition of corn oil leaded to the maximum level of triterpenoid content (Figure 1). The highest level of unsaturation and rich ingredients were observed in corn oil. Some of them or their combination might promote the triterpenoid accumulation. All tested vegetable oils are mainly comprised of oleic acid and linoleic acid, which are long chain fatty acids [33]. Consequently, all tested oils show the positive impacts on both the mycelia growth and the triterpenoid formation. However, our result is completely opposite to a previous study, which indicated that growth of *C. militaris* mycelia was inhibited by linoleic acid during submerged culture [34]. The elimination of the suppression may be due to *A. cinnamomea* being able to make better use of linoleic acid. The assumption is also supported by the facts that *A. cinnamomea* presents different morphological changes. Observation indicates that the addition of corn oil changes the microstructural behavior of submerged *A. cinnamomea* mycelia, which spread out to form more small hyphae than those in the control (Figure 3). That proves that *A. cinnamomea* mycelia grow faster in the presence of corn oil.

The annotations of bio-information increase the current understanding of the regulatory functions of the stimulus from vegetable oil on specific processes, functions, and pathways in *A. cinnamomea*. The metabolic pathways including glycolysis/gluconeogenesis and propanoate metabolism are significantly changed after the treatment of corn oil (Figure 5). That suggests that two pathways may play a role in the improved fermentation of *A. cinnamomea* mycelia by corn oil.

Glycolysis/gluconeogenesis is a crucial central pathway to provide energy and biochemical precursors for living organisms [35]. By KEGG analysis, 4 DEGs (ACg006871, ACg008931, ACg005832, and ACg008798) and 3 DEGs (ACg005832, ACg004264, and ACg004204) are included in glycolysis/gluconeogenesis pathway and propanoate metabolism pathway, respectively. Except ACg004204, all DEGs above are significantly downregulated by the addition of corn oil (Table 1). That indicates corn oil may limit the excessive consumption of central intermediates, such as Acetyl-CoA, in the pathways required for metabolism of non-fermentable carbon sources, energy and the synthesis of many cellular constituents [35,36]. Consequently, the triterpenoid biosynthesis may be enhanced due to more available central intermediate as substrates. In addition, upregulated ACg004204, encoding a lipase (Table 1), possibly activates the hydrolyzation of triglycerides in corn oil to produce more free fatty acids, which improve the mycelium growth and triterpenoid biosynthesis. Those results probably explain that after day 12, mycelial biomass and triterpenoid content still keep a continuous increased trend while the control is significantly inhibited (Figure 4).

Fatty acids are able to modify the cell membrane to increase the permeability and further enhance the biopolymer production [34]. It is possibly because the increasing permeability promotes the absorption of nutrients and the effluxes of metabolites that accelerate the living activity and decrease the toxicity of metabolites for the mycelial cell [25].

**Figure 6:**
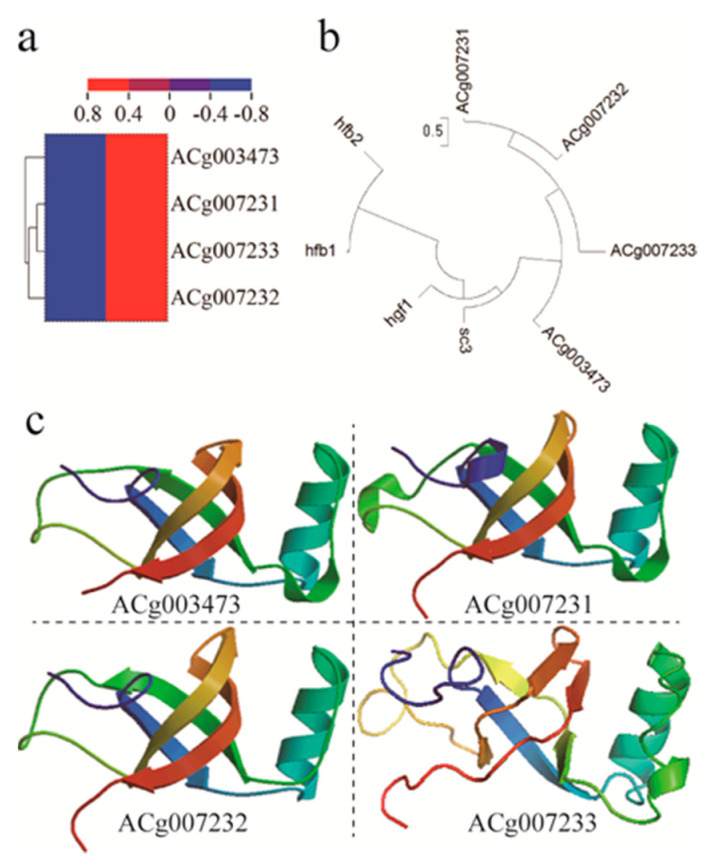
(**a**) A heatmap about differential expressions of four hydrophobin genes. (**b**) Their genetic relation with type I and type II was described by cluster analysis of amino acid sequences. (**c**) Their protein structure was also described using the SWISS-MODEL.

It is worthy to note that the transmembrane hydrophobin pathway happens to change after the treatment of corn oil. That also plays a function on the modification of the mycelial cell membrane. Hydrophobins are a group of small (<20 kDa), highly surface-active globular proteins which play diverse roles in filamentous fungi growth and development [37,38]. Hydrophobins are structurally characterized with four disulfide bonds formed by eight highly conserved cysteine residues in a specific primary sequence pattern [39,40]. Those disulfide bonds stabilize an amphipathic tertiary structure imparting surfactant-like activity, driving hydrophobin self-assembly into amphipathic layers at hydrophobic-hydrophilic interfaces [35,41,42]. In the present study, four hydrophobin genes, including ACg003473, ACg007231, ACg007232, and ACg007233, are identified. They own eight highly conserved cysteine residues and included ~ 100 amino acids (Table S2) in total. By RNA-seq, they are demonstrated to show significant upregulation by more than double (Figure 6a). By cluster analysis using amino sequencing, these four hydrophobins belong to the class I type, like SC3 and HGFI (Figure 6b). We also predicted their crystal structure in the SWISS-MODEL database (Figure 6c), and they almost exhibited a similar structure to templates. These results suggested hydrophobins play a role in improving the liquid fermentation of *A. cinnamomea* by corn oil. One possible mechanism was that the amphipathic layers induced by hydrophobins may contribute to the transportation of corn oil into *A. cinnamomea* mycelial cells.

## 5. Conclusions

In this study, the addition of vegetable oil significantly improved the productivity of the submerged *A. cinnamomea* mycelia in liquid broth. Remarkably, corn oil exhibited the best effects. After a series of optimizations, triterpenoid yield eventually was increased by 5.6-fold, reaching 532.3 mg L^−1^. Differential transcriptome analysis indicated that those improvements were highly relevant to the increasing amounts of hydrophobins and the changes of the primary carbon metabolism.

## Figures and Tables

**Table 1:** DEGs relevant to glycolysis/gluconeogenesis, propanoate metabolism and fatty acids metabolism.

Gene Name	Log2 (FC ^1^)	*p* Value	Annotation
ACg006871	−1.46	1.14E-119	Alcohol dehydrogenase GroES-like domain
ACg008931	−1.64	1.24E-114	Sugar (and other) transporter
ACg005832	−1.29	2.55E-82	Acetyl-CoA synthetase
ACg008798	−1.18	1.15E-54	Mannitol-1-phosphate dehydrogenase
ACg008931	−1.64	1.24E-114	Sugar (and other) transporter
ACg004264	−1.06	9.58E-56	Alanine-glyoxylate amino transferase 2
ACg004204	1.20	6.76E-52	Lipase, hormone-sensitive

^1^ FC indicates fold change.

## Data Availability

All raw sequence reads have been deposited in the NCBI SRA database and are accessible through SRA accession number PRJNA739559 (21 June 2021).

## Supplementary Materials

The following are available online at www.mdpi.com/article/10.3390/jof7110926/s1, Table S1: Differential gene expression analysis under oil treatment; Table S2: Amino acid sequences of hydrophobins.
