# Supplementary material for: Addition of Vegetable Oil to Improve Triterpenoids Production in Liquid Fermentation of Medicinal Fungus Antrodia cinnamomea
Source: J Fungi (Basel). 2021 Oct 31;7(11):926. doi: 10.3390/jof7110926 (PMC8622282; doi:10.3390/jof7110926)
Supplement: Supplementary file 1 [file jof-07-00926-s001.zip › jof-1433439-SI.pdf]

Supporting Information

Addition of vegetable oil to improve triterpenoids production in liquid fermentation of medicinal fungus *Antrodia cinnamomea*

Biaobiao Luo[1, †], Linghui Meng1[, †], Yang Yang[1], Mohammad Omar Faruque[2], Jiuliang Zhang[3], Xiaohua Li[1], and Xuebo Hu[1], [*]

[1]Laboratory of Natural Medicine and Molecular Engineering, College of Plant Science and Technology, Huazhong Agricultural University, Wuhan, 430070, China

[2]Ethnobotany and Pharmacognosy Laboratory, Department of Botany, University of Chittagong, Chittagong, Bangladesh, 4331, Bangladesh

[3]Department of Food and Nutrition, College of Food Science and Technology, Huazhong Agricultural University, Wuhan, 430070, China

[†]These authors contribute equally to this work

[*]Correspondence: xuebohu@mail.hzau.edu.cn; Tel.: 0086-180-6250-3982

Content

Table S1 Page S3-S5

Table S2 Page S6

Table S1. Differential gene expression analysis under oil treatment

GeneID	Diff	log_2FC	P value	Annotation
ACg009074	Up	2.4764384	6.24E-41	*N/A*
ACg008441	Up	1.9032387	2.52E-263	MFS multidrug transporter
ACg007137	Up	1.8273310	2.91E-151	FAD-binding domain
ACg007232	Up	1.3742482	6.09E-94	*N/A*
ACg006971	Up	1.3313856	3.35E-65	*N/A*
ACg003473	Up	1.0991426	1.23E-59	Contributes to surface hydrophobicity, which is important for processes such as association of hyphae in reproductive structures, dispersal of aerial spores and adhesion of pathogens to host structures
ACg007233	Up	1.2536028	3.77E-59	*N/A*
ACg007080	Up	1.0477856	2.52E-58	Glycosyl hydrolase family 71
ACg007231	Up	1.2236576	3.43E-57	*N/A*
ACg004204	Up	1.1993258	5.50E-54	Lipase, hormone-sensitive
ACg001758	Up	1.8775421	1.19E-51	Transporter
ACg008756	Up	1.3238808	4.06E-48	Riboflavin aldehyde-forming enzyme
ACg008408	Up	1.0155715	1.30E-45	Isoprenylcysteine carboxyl methyltransferase (ICMT) family
ACg007935	Up	1.1143509	3.24E-43	Glycoside hydrolase family 18
ACg002304	Up	1.3127590	4.13E-31	DnaJ
ACg001962	Up	1.0074369	5.82E-24	*N/A*
ACg001023	Up	1.1127260	2.44E-18	Major royal jelly protein
ACg007004	Up	1.1074562	6.39E-06	*N/A*
ACg000642	Up	1.1132242	6.93E-04	*N/A*
ACg005259	Up	1.3883839	1.24E-02	*N/A*
ACg006389	Up	4.2664892	1.24E-02	*N/A*
ACg000159	Down	-2.6447621	2.67E-39	GMC oxidoreductase
ACg005309	Down	-1.9272622	6.61E-214	Flavin-binding monooxygenase-like
ACg004077	Down	-1.7686331	3.41E-213	Putative cyclase
ACg008952	Down	-1.9389313	2.19E-190	Occurs in almost all aerobically respiring organisms and serves to protect cells from the toxic effects of hydrogen peroxide
ACg008851	Down	-1.9482786	5.87E-179	Inherit from NOG: Conserved hypothetical, protein
ACg005974	Down	-2.0091623	8.64E-171	Transporter
ACg005318	Down	-1.8888673	2.36E-161	Alpha-glucosidase
ACg003983	Down	-1.6388108	1.49E-159	*N/A*
ACg001915	Down	-2.0403733	1.73E-159	Tripeptidyl peptidase
ACg001732	Down	-2.0431351	3.41E-150	*N/A*
ACg004524	Down	-1.4515484	1.32E-136	Glucosylceramidase
ACg008852	Down	-2.0514382	6.34E-134	*N/A*
ACg003815	Down	-1.3891564	2.16E-133	Carboxylesterase family

ACg001538	Down	-1.5607301	4.12E-133	Acetohydroxy acid isomer reductase, catalytic domain
ACg008856	Down	-1.9944572	1.75E-132	*N/A*
ACg008849	Down	-2.4582420	1.28E-125	Tryptophan dimethylallyl transferase
ACg006871	Down	-1.4637502	2.77E-122	Alcohol dehydrogenase GroES-like domain
ACg007557	Down	-1.3410480	3.12E-121	Chitinase
ACg002632	Down	-1.4393805	3.10E-119	Protein of unknown function (DUF3421)
ACg008931	Down	-1.6389817	3.47E-117	Sugar (and other) transporter
ACg002220	Down	-1.6840855	1.27E-116	Sugar (and other) transporter
ACg002874	Down	-2.0513526	1.60E-112	*N/A*
ACg007041	Down	-1.3149942	6.83E-112	Alpha/beta hydrolase fold
ACg004095	Down	-1.7989585	3.33E-108	Potassium voltage-gated channel, shaker-related subfamily, beta member
ACg004345	Down	-1.3329592	1.01E-95	carnitine
ACg008854	Down	-1.2516958	1.39E-91	Mono-carboxylate
ACg000818	Down	-1.2058513	3.03E-91	Short chain dehydrogenase
ACg001328	Down	-1.5625523	4.90E-90	Glycoside hydrolase family 5
ACg000668	Down	-1.1708956	2.17E-86	Sugar (and other) transporter
ACg005777	Down	-1.3716691	9.59E-86	Catechol dioxygenase
ACg005832	Down	-1.2883867	1.08E-84	Synthetase
ACg005818	Down	-1.1699218	1.80E-83	*N/A*
ACg005663	Down	-1.4005451	2.49E-78	AMP-binding enzyme
ACg004913	Down	-1.1310721	1.73E-77	Phenol 2-monooxygenase
ACg003553	Down	-2.1912079	4.11E-76	L-fucose permease
ACg007125	Down	-1.3822664	3.11E-74	Sterol 24-c-methyltransferase
ACg006921	Down	-1.1075541	6.97E-73	HMG (high mobility group) box
ACg003531	Down	-1.0530572	7.97E-72	*N/A*
ACg003902	Down	-1.3584996	1.72E-71	Short chain dehydrogenase
ACg007556	Down	-1.0857264	3.90E-70	LysM domain
ACg007106	Down	-1.0456316	4.17E-69	Mitochondrial
ACg000575	Down	-1.3913229	6.43E-69	*N/A*
ACg006920	Down	-1.0155080	2.26E-67	Arginyl-tRNA synthetase
ACg007009	Down	-1.2633044	2.31E-66	*N/A*
ACg001117	Down	-1.2774508	1.24E-65	Domain protein
ACg002692	Down	-1.0162451	4.17E-65	Dienelactone hydrolase family protein
ACg003331	Down	-1.6113675	7.12E-64	*N/A*
ACg002589	Down	-1.2067805	1.76E-63	Disrupter of telomere silencing protein Dot5
ACg004240	Down	-1.4517098	4.14E-62	Inherit from fuNOG: NF-X1 finger and helicase domain protein
ACg004264	Down	-1.0622700	7.10E-58	Alanine--glyoxylate aminotransferase 2
ACg008798	Down	-1.1849239	8.83E-57	Mannitol-1-phosphate dehydrogenase
ACg003543	Down	-1.1426180	9.50E-56	Acyl-CoA dehydrogenase, middle domain
ACg000540	Down	-1.2463001	1.04E-55	*N/A*
ACg008853	Down	-1.2468301	7.70E-55	Cytochrome P450

ACg004062	Down	-1.1058458	1.51E-47	BTB/POZ domain
ACg003358	Down	-1.1853109	1.78E-47	Hydrolase family 10
ACg007894	Down	-1.0622058	1.95E-47	Cytochrome p450
ACg002169	Down	-1.1017845	4.04E-47	Sugar (and other) transporter
ACg002889	Down	-1.0743323	6.82E-47	Alpha beta hydrolase
ACg005148	Down	-1.6833600	1.59E-46	*N/A*
ACg000141	Down	-1.2171070	2.80E-46	Flavin containing amine oxidoreductase
ACg002089	Down	-1.0318278	6.50E-46	Acyl-CoA dehydrogenase, N-terminal domain
ACg008857	Down	-2.5154666	8.18E-45	*N/A*
ACg006199	Down	-1.0017287	4.59E-43	Protease, serine, 16 (thymus)
ACg001118	Down	-1.0395905	4.13E-39	Domain protein
ACg000529	Down	-1.0253118	4.98E-37	*N/A*
ACg006397	Down	-1.2844515	5.30E-37	Major Facilitator Superfamily
ACg005648	Down	-1.1033594	1.37E-36	Trehalase
ACg000195	Down	-1.0828408	6.82E-36	*N/A*
ACg002633	Down	-1.1709579	4.29E-35	Receptor
ACg006019	Down	-1.2800294	5.39E-33	*N/A*
ACg001071	Down	-1.5184314	8.51E-33	*N/A*
ACg002511	Down	-1.0736893	3.18E-27	*N/A*
ACg001964	Down	-1.8960253	3.73E-27	Inherit from fuNOG: fumarylacetoacetate hydrolase
ACg002896	Down	-1.1677912	4.34E-23	*N/A*
ACg002895	Down	-2.0335373	2.85E-21	*N/A*
ACg000821	Down	-1.3903883	7.63E-20	Short chain dehydrogenase
ACg006829	Down	-1.3909792	8.93E-20	Sugar (and other) transporter
ACg005150	Down	-1.2701456	1.92E-19	*N/A*
ACg000075	Down	-1.1377992	5.60E-19	Sugar (and other) transporter
ACg008451	Down	-1.0639441	2.17E-18	*N/A*
ACg000925	Down	-1.4204323	1.88E-16	*N/A*
ACg006427	Down	-1.2455964	3.16E-16	GDSL lipase acylhydrolase family protein
ACg003097	Down	-1.0146153	2.45E-12	Heat shock protein
ACg008090	Down	-1.0354521	1.02E-08	*N/A*
ACg008447	Down	-1.0445379	3.75E-05	Caspase domain
ACg008767	Down	-1.3394176	1.80E-04	*N/A*
ACg009239	Down	-2.0768630	5.97E-04	Inherit from COG: Retrotransposon protein
ACg000050	Down	-1.7330041	6.68E-04	*N/A*
ACg000808	Down	-1.8046440	3.57E-03	*N/A*
ACg005753	Down	-1.1933009	5.53E-03	*N/A*
ACg006627	Down	-4.0019067	5.96E-03	Inherit from fuNOG: reverse transcriptase
ACg003560	Down	-4.3256940	9.48E-03	*N/A*

Table S2. Amino acid sequences of hydrophobins

Gene	Amino acid sequence
ACg003473	MMFRTVLVLAFALSALSAPQSSSSQCDSGTIQCCDTVAPAYSASVAPVLKALKINVDDENKQVALGCGGSAVGVGAGSSCTSAPICCENNYFGLIGIGCISITL
ACg007231	MMFRAVTFLTFALSALAVPQAPYVDQCNPGSVLCCDSVYSPSSVYATSLVKALQIDNVPQDKQIATGCGGGGINVGGGSTCNSNPVCCERDGLGGLIGIGCAAIPVSI
ACg007232	LRNPFILPSRTAIRTPSYIALSSHQAHRPLSFTIMMFRAVALLTFALSALAVPQYGPTSQCDSGSELCCDSVYSPGTVYATSLIKALNIDNVPVGHIATGCGGGGVNVGGGSTCNTNPVCCQGNALGGLIGVGCAAIPVSI
ACg007233	MRLSLSLLSLALPLLALAAPQTFGPGPQCDGGPVECCDATYRADSPEGSAIMKAMKIDARNVTGSTIIAAACGGGGVNVGGGSTCNNIPVCCNNTYFGLIGVGCASIPVSI
